# Chemical composition and insecticidal potential of botanical fractionation extracts for the management of *Sitophilus zeamais* Motschulsky, 1855 (Coleoptera: Curculionidae) in stored maize

**DOI:** 10.1016/j.heliyon.2025.e42131

**Published:** 2025-01-21

**Authors:** Fikadu Kifle, Melaku Girma, Araya Gebresilassie, Yitbarek Woldehawariat, Estifanos Ele

**Affiliations:** a College of Natural and Computational Sciences, Department of Biology, Hawassa University, P.O. Box 05, Hawassa, Ethiopia; b College of Natural and Computational Sciences, Department of Zoological Sciences, Addis Ababa University, P.O. Box 1176, Addis Ababa, Ethiopia; c College of Natural and Computational Sciences, Department of Chemistry, Addis Ababa University, P.O. Box 1176, Addis Ababa, Ethiopia

**Keywords:** Chemical composition, Fractionated extract, Insecticidal potential, Maize weevils, *Sitophilus zeamais*

## Abstract

The weevil *Sitophilus zeamais* (Motschulsky, 1855) (Coleoptera: Curculionidae) is a destructive pest of stored maize, particularly in warm and humid tropical regions. The objective of this study was to analyze the chemical composition and evaluate the insecticidal potential of fractionated extracts of *Eucalyptus globulus*, *Tagetes minuta* and *Annona squamosa* used as natural alternatives to synthetic insecticides in the management of *S. zeamais*. Fractionated extracts were obtained by macerating and soaking *E. globulus* leaves, whole *T. minuta* plants and *A. squmosa* seeds using hexane, chloroform, and methanol as extraction solvents. Experiment tests were carried out at different doses (5, 10, and 15 g/kg) on adult *S. zeamais* to investigate adult mortality rate, progeny inhibition rate, repellent effect, fumigation effects, and effects on grain weight loss. Mortality of adult *S. zeamais* was observed after application at 24, 48, and 98 h. All fractions exhibited insecticidal activity, which increased with higher doses and longer post treatment periods. At a dose of 15 g/kg, the hexane, chloroform and methanol fractions of *E. globulus* caused a mortality rate of 100 % at 48 h of exposure and an inhibition rate of more than 100 % in progeny production. All fractions at lower doses (5 g/kg) showed a lower mortality of *S. zeamais*, which was significantly less than that of Actellin 2 % dust used as a positive control. The composition of bioactive extracts was analyzed using a gas chromatography-mass spectrometry (GC-MS) apparatus. GC-MS results of the major components of the *E*. *globulus* extract were aromandendrene (24.83 %), globulol (10.33 %), heneicosane (15.56 %), hexadecanoic acid, and methyl ester (7.50 %). For *A. squamosa*, there was nonadecane (2.35 %), hexadecanoic acid methyl (16.06), heneicosane (25.42 %), 9, 12-octadecadienoic acid (23.35 %), and 9-octadecenoic acid (26.466 %), and methyl stearate (2.55 %). The major components identified from the fractionated extract of *Tagetes minuta* were methyl 10-trans, linolenic acid (62.79 %), hexadecanoic acid, methyl ester (19.10 %) and heneicosane (5.78 %), and methyl stearate (5.46 %). *E. globulus* leaves and their constituent compounds have potential for the development of natural insecticides or fumigants for the control of maize weevils in stored grains. The findings can contribute to developing eco-friendly, safer and more sustainable alternatives to synthetic chemical insecticides.

## Highlights

• The findings support the use of plants extracts as natural insecticides in stored grain management.
• Linoleic acid, Aromandendrene and 12-Octadecadienoic acid are the major active compounds in the *Eucalyptus globulus* leaves, whole plant of *Tagetes minuta*, and seeds of *Annona squmosa* extracts, respectively and evaluating their efficacy against this major storage pest, the research provide sustainable and eco-friendly alternatives to synthetic insecticides, contributing to improved post-harvest pest management practices.
• Minimizing harm to the environment, promoting sustainable practices, reducing chemical residues, and offering safer alternatives for human and animal health.
• Further research is recommended to optimize extraction methods and assess long-term efficacy and safety.

## 1 Introduction

The weevils *Sitophilus zeamais* (Motschulsky, 1855) (Coleoptera: Curculionidae), commonly known as the maize weevils or the corn weevil, is a significant pest that affects maize grains [1,2]. It is a small beetle. belonging to the family Curculionidae [3]. The maize weevil is native to Central America has spread to various regions throughout the world due to global trade and transport [2,4,5].

The impact of *S. zeamais* on maize grains is substantial for farmers in agriculture, agricultural industry, and economic terms [6]. The maize weevils primarily infest stored maize grains [7,8]. The infestation can occur during storage and transportation or even in the field if favorable conditions exist [7,9]. Adult beetles lay eggs inside the grains, and the larvae develop and feed within the kernels. Weevils consume and destroy a considerable portion of stored maize, making it unsuitable for human or animal consumption [10]. The feeding activity of larvae creates openings in the kernels that allow various fungi, molds, and bacteria to enter [11]. These secondary infections can cause further deterioration of grain quality, produce mycotoxins, and increase the risk of human and animal health issues [12]. Infestation by *S. zeamais* can lead to significant grain losses in storage [13].

Controlling maize weevil infestations poses challenges for farmers and storage facilities. Weevils are resilient and can develop resistance to chemical insecticides over time [11,14,15]. In addition to their environmental impacts, synthetic insecticides are not always easily accessible to farmers, especially in developing countries. This is because synthetic insecticides can be expensive and farmers may not have access to the necessary equipment to apply them safely. Effective and eco-friendly control is required due to their ability to hide inside seeds and adapt to a wide range of environmental conditions [7].

Natural plant-based pest control offers an effective way to manage pests while minimizing environmental harm, supporting sustainable practices, reducing chemical residues, and providing safer alternatives for human and animal health [11,[16], [17], [18]]. In a preliminary study, several plant species were evaluated for their insecticidal potential and three of these species were selected for further analysis.

Therefore, this study aimed to analyze the chemical composition using gas chromatography-mass spectrometry (GC-MS) on fractionated extracts of *E. gobulus, T. minuta,* and *A. squamosa* in hexane (apolar), chloroform (intermediate), and methanol (polar) solvents and to test their insecticidal potential on maize against *S. zeamais* infestation.

## 2 Materials and methods

### 2.1 Insect rearing

Adult *S. zeamais* was reared in the Entomology Research Laboratory of the Department of Zoological Sciences at Addis Ababa University. The experiment was conducted under controlled conditions, with a temperature of 27 ± 5 °C and a relative humidity of 70 ± 5 %. Ten kilograms of the Awash Q6 maize seed variety were obtained from the Melkasa Agriculture Research Center. The seeds were cleaned to remove any visible damage and disinfected by washing with deionized water, sterilizing in 70 % ethanol, and freezing for two weeks at temperatures between −20 °C and 0 °C to eliminate internal infestations [9]. To acclimatize, the seeds were stored in a plastic jar and kept under experimental conditions for at least two weeks [15].

A maize weevil culture was established to provide uniformly aged weevils for the experiment. Insects were placed in plastic jars and covered with muslin cloth secured by rubber bands to allow ventilation while preventing insect escape [3]. After two weeks, all adult weevils, both alive and dead, were counted and removed. The study utilized newly emerged unsexed adult *S. zeamais* aged three to ten days. The sexes of the weevils were identified using a hand lens by examining their snouts: females have longer, thinner snouts with smooth-textured bodies, while males have shorter, thicker snouts with rougher textures [6].

### 2.2 Collection of botanicals

Based on the researcher's recommendations and personal observation in the community, *Eucalyptus globulus* (leaves), marigold (*Tagetes minuta*) (whole plant), and custard apple (*Annona squamosa*) (seed) were selected against *S. zeamais*. similarly, in the preliminary study, they were screened. All parts of plants were collected from the surrounding areas in Addis Ababa city, Ethiopia, as well as Actellic dust 2 %, which was purchased from the local market in Merekato, Addis Ababa and utilized as the standard check. Untreated grain was used as a control for comparison. The leaves of *E. globulus*, *A. squamosa* seed and whole plants of *T. minuta* were dried in the shade. The dried leaves and seeds of the plants were ground into a fine powder using a mortar and pestle [11,16].

### 2.3 Botanical extraction

The dry powders (400 g) were placed in 750 ml solvents within a 2000 ml round-bottom flask. Hexane (nonpolar), chloroform (intermediate) and methanol (polar) were used as solvents. The plant's powder was soaked in solvent and macerated on a shaker bath in the chemistry laboratory for 48 h. It was then filtered using Whatman No. 8 filter paper and a funnel. The residue was re-filtered and dried in the laboratory for 10 h at room temperature (24 °C). Then, the residue gained from hexane was extracted using chloroform, followed by methanol. This was done in an attempt to extract the entire active and soluble chemicals present using the three solvents. The filtrates obtained with hexane, chloroform, and methanol were concentrated separately using rota vapour (Heidolph Rotavac valve control, Germany) at 50 °C at 120 rpm. The same extraction process was performed for all plant species. After complete evaporation of the solvents, the different fractions of the extracts were weighed, stored in airtight containers and kept in a refrigerator at −4 °C until needed for the experiments.

### 2.4 *Sitophilus zeamais* mortality rate

Three different concentrations of the extracts (5, 10, and 15 g/kg of grain prepared from 1 g of w/w + 1 ml of respective solvents) were added separately into 100 g of maize within 500 ml glass jars and manually stirred for 4 min. The jars were then left exposed to air in the laboratory for 45 min to allow complete evaporation of the solvents. The active ingredient of Actellic dust 2 % is pirimiphos-methyl at a concentration of 2 %, used as a standard check while untreated seeds were used as a control. After complete evaporation, a group of 20 unsexed insects [20] aged between three to seven days were added to each plastic jar and covered with a muslin cloth to allow air circulation. Each treatment was repeated three times. The mortality rate was evaluated after 24, 48, and 98 h. The calculation of the mortality rate was corrected for control mortality according to Abbott's formula [21]:

$$\text{MC}=\frac{\text{Mo}-\text{Mc}}{100-\text{Me}}\;x100$$

Where; Mo = observed mortality rate of treated (%), Me = control mortality rate (%), and Mc = corrected mortality rate (%).

### 2.5 F1 progeny test

The treated jars were kept for an additional 10 days of oviposition time after the mortality assessment. All living and dead insects were removed. The glass jars were then sealed and kept under the same ambient conditions for the assessment of the F1 progeny [22]. The recording of F1 progeny was conducted once a week for six weeks. To determine which treatment inhibited the emergence of F1 progeny, the percentage reduction in adult emergence, or inhibition rate (% IR), was calculated, following the methods used by Tapondjou et al. [23].

$$\text{IR}\%=\frac{\text{Total}\;F1\;\text{progeny}\;\text{in}\;\text{control}-\text{Total}\;F1\;\text{progeny}\;\text{in}\;\text{treatment}}{\text{Total}\;F1\;\text{progeny}\;\text{in}\;\text{control}}x100$$

Where: IR: Inhibition Rate.

### 2.6 Repellency test

**Fig. 1:**
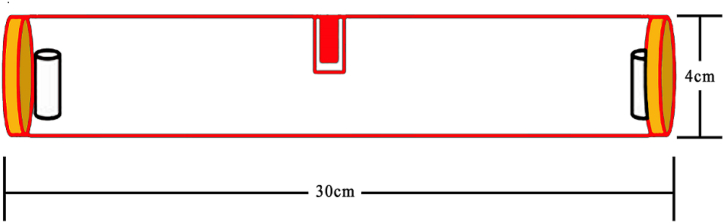
Linear olfactometer set-up for repellency test. a). insect entrances and b and c).sample container.

The repellent activities of fractionated extracts were investigated using a method followed by Refs. [24,25], which used a linear olfactometer for the experiments. The linear olfactometer consisted of a 30 cm glass tube with a diameter of 4 cm and a 29 mm hole in the middle (Fig. 1). At each end of the tube, a small container was placed. One container contained 10.0 g of untreated maize, while the other container contained 10.0 g of maize seeds mixed with a fractionated extract solution at concentrations of 0.1 %, 0.5 % and 1 %. The hole in the middle of the tube was covered to prevent insect escape, and the ends of the tube were sealed with plastic Petri dishes. Twenty adult *S. zeamais* insects were released into the middle of the tube through the hole. The behavior of the insects was observed and their positions were recorded for 25 min. The insects that have made a choice are those that have entered one of the containers containing the maize or those that have reached the last 14 cm or the end of the tube. For those who chose the middle of the tube, some insects made their choice as time passed.

The percentage repellence was calculated using the formula cited in Ref. [25]. PR =2X(C−50)

where: C is the percentage of insects in the negative control bottle. The results were interpreted following the scale by McDonald et al. [26] as follows in Table .1.

**Table 1:** Repellency scale from the least to the most repellent, 0 to V.

Class	Repellence rate%	Interpretations
0	>0.01 to< 0.1	none repellence
I	0.1 to 20	very weakly repellent
II	20.1 to 40	Moderately repellent
III	40.1 to 60	Averagely repellent
IV	60.1 to 80	Fairly repellent
V	80.1 to 100	Very repellent

### 2.7 Fumigation test

**Fig. 2:**
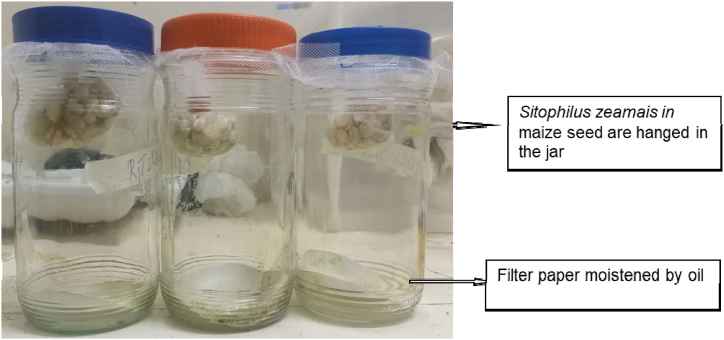
Experimental set-up for Fumigation test.

The fumigant toxicity of the fractionated extract was evaluated using a modified version of the [27] method. One-liter wide-mouth bottles with lids were used (Fig. 2). Filter papers of 8 mm diameter were treated with 1, 2, and 3 ml of fractionated extracts at rates of 12 mg, 60 mg, and 300 mg diluted in 10 ml of deionized water; the same amount of neem crude extract served as a control. After allowing the solvent to evaporate for 20 min, the filter paper was placed at the bottom of a 1-liter glass bottle [27]. Twenty insects in a small nylon mesh bag with 100 g of seed were hung in the center of the glass bottle (7 cm high) above the filter paper (Fig. 2). The bottles were then closed tightly with a lid. Each treatment was replicated three times. Mortality was checked after 24 h and calculated as follows.

$$\text{Weevils}\;\text{Mortality}\%=\frac{Numbers\;of\;dead\;weevils}{Total\;numbers\;of\;weevils}x100$$

### 2.8 Gas chromatography mass spectrometry (GC-MS) analysis

GC-MS analysis on fractionated hexane extracts obtained from *E. globulus* leaves, *A. squmosa* seed and the whole plant of *T. minuta* was made using an Agilent Technologies G4567A GC system coupled with Agilent Technologies G4567A MSD system. The chromatographic separation was conducted on a fused silica capillary column, DB-1701, with a 30 m length, 0.25 mm inner diameter and 0.25 μm film thickness at a pressure of 8 psi and a flow rate of 1 mL/min. Ultra-high pure helium (99.999 %) was used as carrier gas at constant flow mode. An Agilent G4567A autosampler was used to inject 1 μL of the sample with a splitless injection mode into the inlet heated to 275 °C with a total run time of 29.33 min. The oven temperature was programmed with the initial column temperature of 60 °C and a hold-time of 2 min. The column temperature was increased at a rate of 10 °C/min until the temperature reached 200 °C and then heated again at the rate of 3 °C/min until the temperature reached 24 °C. No mass spectra were collected during the first 4 min of the solvent delay. The transfer line and the ion source temperatures were 280 °C and 230 °C, respectively. The detector voltage was 1600 V and the electron energy was 70 eV. Mass spectral data were collected from 40 to 650 *m*/*z*. The names, structures, and qualities of peaks were determined through a NIST 2014 library search. The relative percentage of the chemical constituents present in the extracts of the hexane fraction obtained from *E. globulus, A. squmosa* seed, and the whole plant of *T. minuta* was determined by normalization of the peak area.

### 2.9 Data analysis

The experiments were carried out in a completely randomized design (CRD). All data entry and analysis were performed using Microsoft Excel, JMP Pro 19, SAS Institute, Inc., and R-software. To find out the effects of treatment on % mortality and % number of F1 progeny, the repellence test and fumigation test were performed with factorial analysis and two-way ANOVA analysis. In cases, where significant results were obtained, mean comparisons were made using Tukey's standardized range test at a p < 0.05 level of significance.

## 3 Results

### 3.1 Effect of plant extracts on insect's mortality

**Fig. 3:**
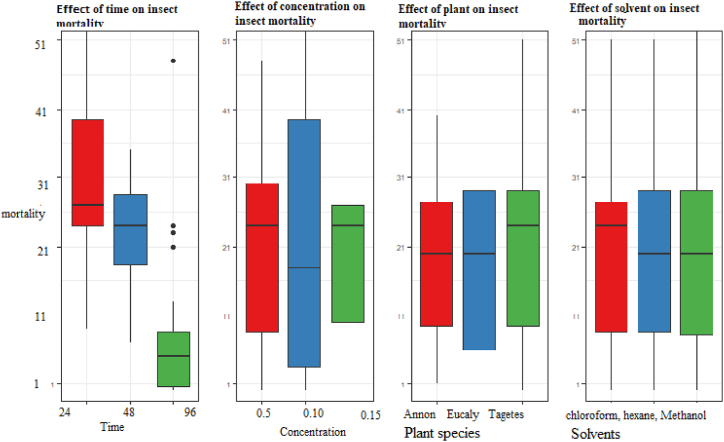
Effect of plant species, time, concentration and solvent on the mortality of *Sitophilus zeamais*.

The results on the mortality rate of adult *S. zeamais* starting from 24, 48 and 96 h after introduction to the seed mixture with different fractionated extracts are shown in Fig. 3. At high concentration rate (15 g/kg) of hexane, chloroform and methanol extracted from *E. globulus* were recorded (85.00 %, 85.00 %, and 88.00 %) of adult *S. zeamais* mortality at 24 h after treatment application, respectively. At a high concentration rate (15 g/kg), *T. minuta* fraction extracts of hexane, chloroform, and methanol were recorded (83.00 %, 83.00 %, and 85.00 % of adult mortality of *S. zeamais* at 24 h after treatment application, respectively), while at a high concentration rate (15 g/kg), *A. squmosa* fraction hexane, chloroform, and methanol were recorded at 65.00 %, 58.33 %, and 66.66 % of adult mortality of *S. zeamais* at 24 h after treatment application, respectively (Table 3).

**Table 2:** Factorial two-way ANOVA in the main effects of extracts *Eucalyptus globulus leaves, Tagetes minuta,* and *Annona squmosa* on mortality of adult *Sitophilus zeamais* after application.

Response	df	Sum. square	Mean Square
Time	2	7833.7	3916.8∗∗∗
Plant Species	2	90.5	45.3 ns
Solvent	2	33.1	16.5 ns
Concentration	2	50.4	25.2 ns
Time v/s Concentration	4	4407.9	1102∗∗∗
Plant Species v/s Solvent v/s concentration	4	70.3	17.6 ns
Residuals	64	4157.4	4157.4

df = degree of freedom, ns = non-significant.

**Table 3:** Combined effect of mortality mean % ±SD of adult *Sitophilus zeamais* due to treatment of maize seeds with fractionated extract, after a different exposure time and solvents.

Plant Species	Dose	adult mortality at 24 h			Adult mortality at 48 h			Adult mortality at 96 h		
	g/kg	Hexane	Chloroform	Methanol	Hexane	Chloroform	Methane	Hexane	Chloroform	Methane
*Annona squmosa*	5	31.66 ± 2.31^a^	16.66 ± 1.52^a^	15.00 ± 1.00^a^	54.66 ± 3.21^c^	58.30 ± 1.52^b^	60.00 ± 1.00^ab^	95.00 ± 1.15^bc^	96.60 ± 0.57^cd^	100.00 ± 1.73^d^
	10	28.33 ± 1.53^a^	40.00 ± 1.00^ab^	33.33 ± 1.52^ab^	64.00 ± 1.15^c^	90.00 ± 1.00^bc^	84.95 ± 3.21^bc^	100.00 ± 0.58^d^	100.00 ± 1.73^d^	100.00 ± 2.00^d^
	15	65.00 ± 1.00^c^	58.33 ± 0.5^bc^	66.66 ± 1.52^c^	98.30 ± 0.58^d^	91.6 ± 0.57c	96.65 ± 1.73^cd^	100.00 ± 0.58^d^	100.00 ± 0.57^d^	100.00 ± 0.57^d^
*Eucalyptus globulus*	5	41.66 ± 1.52^bc^	41.66 ± 0.57^bc^	40.00 ± 1.00^b^	88.30 ± 0.57^bc^	91.65 ± 1.00^c^	91.65 ± 1.52^cd^	100.00 ± 1.52^d^	100.00 ± 1.15^d^	100.00 ± 1.52^d^
	10	61.66 ± 3.21^c^	45.00 ± 2.64^bc^	60.00 ± 5.19^c^	93.30 ± 2.08^cd^	86.65 ± 0.57^b^	93.30 ± 4.04^cd^	100.00 ± 1.15^d^	100.00 ± 2.51^d^	100.00 ± 1.15^d^
	15	85.00 ± 7.72^d^	85.00 ± 1.00^cd^	88.33 ± 1.52^cd^	100.00 ± 0.81^d^	100.00 ± 1.00^d^	100.00 ± 1.52^d^	100.00 ± 0.00^d^	100.00 ± 0.00^d^	100.00 ± 0.00^d^
*Tagetes minuta*	5	45.00 ± 1.00^ab^	41.66 ± 1.52^bc^	36.66 ± 2.08^a^	93.30 ± 0.57^cd^	86.65 ± 1.00b^cd^	76.65 ± 3.60^b^	93.30 ± 0.57^bc^	96.65 ± 0.00^cd^	98.30 ± 1.15^d^
	10	50.00 ± 1.00^bc^	53.33 ± 2.30^c^	65.00 ± 1.00^bc^	93.30 ± 0.57^cd^	93.30 ± 3.60^cd^	88.30 ± 2.08^bc^	96.00 ± 0.57^cd^	100.00 ± 1.52^d^	98.30 ± 1.52^d^
	15	83.33 ± 1.00^cd^	83.33 ± 1.52^cd^	85.00 ± 1.00^cd^	100.00 ± 1.15^d^	100.00 ± 1.52^d^	100.00 ± 1.00^d^	100.00 ± 0.00^d^	100.00 ± 0.00^d^	100.00 ± 0.00^d^
Actellic2%dust	5	100.00 ± 0.00^d^	100.00 ± 0.00^d^	100.00 ± 0.00^d^	100.00 ± 1.15^d^	100.00 ± 1.52^d^	100.00 ± 1.00^d^	100.00 ± 0.00^d^	100.00 ± 0.00^d^	100.00 ± 0.00^d^
Control		00.00 ± 0.00	00.00 ± 0.00	00.00 ± 0.00	00.00 ± 0.00	00.00 ± 0.00	00.00 ± 0.00	00.00 ± 0.00	00.00 ± 0.00	00.00 ± 0.00

The means within a column followed by different letters is significantly different, p < 0.05, Tukey studentized range test (HSD).

The efficiency of the extract was significantly different based on the time after application (p < 0.05) (Table 2). Likewise, a statistically significant difference was seen in the interaction between time and concentration. However, the solvent type and concentration rate of plant species showed a non-significant effect on the mortality of *S. zeamais*.

### 3.2 F1- progeny production and inhibition rate

**Fig. 4:**
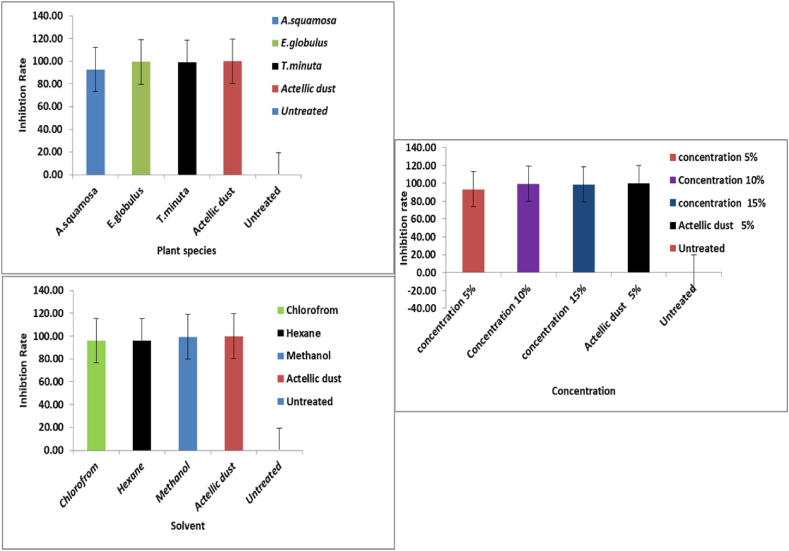
Inhibition rate of insect in seeds treated with fractionated extracts of plant species at different concentration and solvents.

There was a statistically significant difference (p < 0.05) in the number of F1 progeny produced by adult *S. zeamais* on the plant species and concentration. Non-significant difference effect on the interaction between plant species and the solvent type. Similarly, there was a non-significant interaction between the effects of plant species with solvent and treatment vs solvent and concentrations on the seed maize (Table 4). There was a statistically significant difference (p < 0.05) on number of inhibition rates due to the treatment effect on adult *S. zeamais* (Table 5). There was a non-significant interaction between the effect of treatment v/s solvent and treatment v/s solvent v/s concentration (Table 5). The emergence of adult progeny was not observed from Actellin dust 2 % treated maize seed. *E. globulus* and *T. minuta* showed the highest inhibition rate. The inhibition rate of adult S. zeamais was significantly increased when the concentration level increased (Fig. 4).

**Table 4:** Factorial two-way ANOVA in the main effects of extracts *Eucalyptus globulus leaves, Tagetes minuta, and Annona squmosa* on F1 Progeny adult emergence of *Sitophilus zeamais*.

Source	Nparm	df	Sum of Squares	F Ratio	Prob > F
Treatment	2	2	19.43	15.74	<0.0001^a^
Solvent	2	2	4.84	3.92	0.0257^a^
Concentration	2	2	15.51	12.56	<0.0001^a^
Treatment vs Solvent	4	4	5.83	2.36	0.0648 ns
Treatment vs Concentration	4	4	12.94	5.24	0.0012^a^
Solvent vs Concentration	4	4	8.64	3.50	0.0130^a^
Treatment vs Solvent x Concentration	8	8	9.58	1.94	0.0725 ns

a Indicate = significant, df = degree of freedom, ns = non-significant, par = number of parameters.

**Table 5:** Factorial two-way ANOVA in the main effects of extracts *Eucalyptus globulus leaves, Tagetes minuta, and Annona squmosa* on inhibition rate for control of *Sitophilus zeamais*.

Source	Nparm	df	Sum of Squares	F Ratio	Prob > F
Treatment	2	2	781.69	14.2057	<0.0001^a^
Solvent	2	2	194.82	3.5405	0.0359^a^
Concentration	2	2	625.92	11.3750	<0.0001^a^
Treatment v/s Solvent	4	4	231.97	2.1078	0.0925 ns
Treatment v/s Concentration	4	4	531.39	4.8286	0.0021^a^
Solvent v/s Concentration	4	4	358.76	3.2599	0.0182^a^
Treatment v/s solvent v/s Concentration	8	8	402.16	1.8271	0.0919 ns

a Indicate significant difference, df, degree of freedom, Nparm = Number of parameters, ns = non-significant.

### 3.3 Repellency effect

Statistically significance interaction was observed between the treatment of plant species, solvent, concentration, plant species v/s concentration and solvent v/s concentration (p < 0.05) (Table 7). While, has no significance difference interaction between the plant species v/s solvent. Different doses of fractionated extracts (0.1, 0.5 and 1 %) caused 69.23 %–96.61 % repellency for all fraction extracts (Table 6).

**Table 6:** Percentage Repellences of Sitophilus zeamais due to treatment of maize seeds with different concentrations of fractionated extracts of *Eucalyptus globulus* leaves, *Tagetes minuta*, and *Annona squmosa* and *Azadarchita indica* oil as the standard check.

Plant/Extracts	Dose (%)	Repellency rate	Class	Interpretation
*Annona squmosa*/hexane	0.1	75.00	IV	Fairly repellent
	0.5	70.00	IV	Fairly repellent
	1	96.33	V	Very repellent
*Annona squmosa/*chloroform	0.1	69.23	IV	Fairly repellent
	0.5	73.58	IV	Fairly repellent
	1	96.00	V	Very repellent
*Annona squmosa*/methanol	0.1	67.27	IV	Fairly repellent
	0.5	74.55	IV	Fairly repellent
	1	81.13	V	Very repellent
*Eucalyptus globulus*/hexane	0.1	88.46	V	Very repellent
	0.5	92.16	V	Very repellent
	1	96.61	V	Very repellent
*Eucalyptus globulus*/chloroform	0.1	83.78	V	Very repellent
	0.5	92.86	V	Very repellent
	1	96.00	V	Very repellent
*Eucalyptus globulus*/methane	0.1	76.92	IV	Fairly repellent
	0.5	80.23	V	Very repellent
	1	89.74	V	Very repellent
*Tagetes minuta*/hexane	0.1	88.24	V	Very repellent
	0.5	89.23	V	Very repellent
	1	96.08	V	Very repellent
*Tagetes minuta*/chloroform	0.1	69.23	IV	Fairly repellent
	0.5	73.58	IV	Fairly repellent
	1	96.00	V	Very repellent
*Tagetes minuta*/methanol	0.1	67.27	IV	Fairly repellent
	0.5	74.55	IV	Fairly repellent
	1	82.46	V	Very repellent
*Neem oil*	0.1	74.55	IV	Fairly repellent
	0.5	82.46	V	Very repellent
	1	97.45	V	Very repellent

**Table 7:** Factorial two-way ANOVA in the main effects of extracts *Eucalyptus globulus leaves, Tagetes minuta, and Annona squmosa* on repellence rate the control of *Sitophilus zeamais*.

Source	Nparm	df	Sum of Squares	F Ratio	Prob > F
Plant species	2	2	832.00	7.29	0.0016^a^
Solvent	2	2	2549.27	22.34	<0.0001^a^
Concentration	2	2	3335.81	29.24	<0.0001^a^
Plant species v/s Solvent	4	4	214.63	0.94	0.4475 ns
Plant species v/s Concentration	4	4	728.16	3.19	0.0200^a^
Solvent v/s Concentration.	4	4	895.86	3.93	0.0072^a^
Plant species v/s Solvent v/s Concentration	8	8	219.28	0.48	0.8646 ns

a Indicated that significance difference, (df) = degree of freedom, ns = non-significant.

### 3.4 Fumigation toxicity of fractionated extracts tests

The result of the fumigation toxicity of fractionated extracts of *E. globulus* leaves, *T. minuta* and *A. squmosa* towards *S. zeamais* is presented in Table 9. Statistically significance difference detected on the solvent type and concentration (p < 0.05) (Table 8). The highest dose (3 mg) of fractionated extracts showed significantly (p < 0.05) higher mortality of adult *S. zeamais* (Table 8). All treated fractions did not cause significant mortality of *S. zeamais* at lower levels of application (0.12 mg/kg and 0.6 mg/kg). However, of the three fractionated extracts, the fractionated extracts of *T. minuta* recorded the highest mortality with a high concentration of 3 mg at 24 h (Table 9).

**Table 8:** A Factorial two-way ANOVA parameter for main effects and interactions fumigant test counts for *Sitophilus zeamais* mortality.

Source	Nparm	df	Sum of Squares	F Ratio	Prob > F
Plant species	2	2	24.07	0.48	0.6205 ns
Solvent	2	2	235.19	4.70	0.0131^a^
Concentration	2	2	2205.56	44.11	<0.0001^a^
Plant species v/s Solvent	4	4	207.41	2.07	0.0969 ns
Plant species v/s Concentration	4	4	120.37	1.20	0.3199 ns
Solvent v/s Concentration	4	4	209.26	2.09	0.0944 ns
Plant species v/s Solvent v/s Concentration	8	8	653.71	3.27	0.0041^a^

a Indicated that significance difference, (df) = degree of freedom, ns = non-significant.

**Table 9:** Percentage Mean ± SD Fumigation toxicity of three plant extract with different Solvent and control treatments against *Sitophilus zeamais* after 24 h exposure. Means within a column followed by different letters is significantly different, P < 0.05, Tukey studentized range test (HSD).

Treatment	Dose(mg/kg)	Mortality		
		Hexane	Chloroform	Methanol
*Annona squamosa*	0.12	5.00 ± 10.0^a^	1.60 ± 0.57^a^	5.00 ± 10.00^a^
	0.6	6.60 ± 0.57^bc^	6.60 ± 0.57^ac^	13.00 ± 1.52^cd^
	3.0	15.00 ± 10.0^d^	10.00 ± 10.0^c^	20.00 ± 10.0^d^
*Eucalyptus globulus*	0.12	5.00 ± 0.57^a^	5.00 ± 10.0^c^	3.00 ± 0.57^a^
	0.6	11.66 ± 1.00^bc^	1.66 ± 0.57^a^	10.00 ± 2.00^bc^
	3.0	13.33 ± 1.52^bcs^	15.00 ± 10.0^d^	21.66 ± 0.57^d^
*Tagetes minuta*	0.12	00.00 ± 00^a^	0.00 ± 0.00^a^	0.00 ± 00.00^a^
	0.6	10.00 ± 5.00^bc^	15.00 ± 10.00^cd^	5.00 ± 10.00^a^
	3	25.00 ± 1.52^d^	5.00 ± 1.00^a^	15.00 ± 2.65^d^
*Neem oil*	0.12	10.00 ± 2.23^bc^	0.00 ± 0.00^a^	10.00 ± 0.00^bc^
	0.6	15.00 ± 0.00^cd^	15.00 ± 0.00^cd^	10.00 ± 0.00^bc^
	3	30.00 ± 0.00^d^	25.00 ± 0.00^d^	15.00 ± 0.00^d^

### 3.5 GC-MS analysis of *Annona squmos*a seed extract

**Fig. 5:**
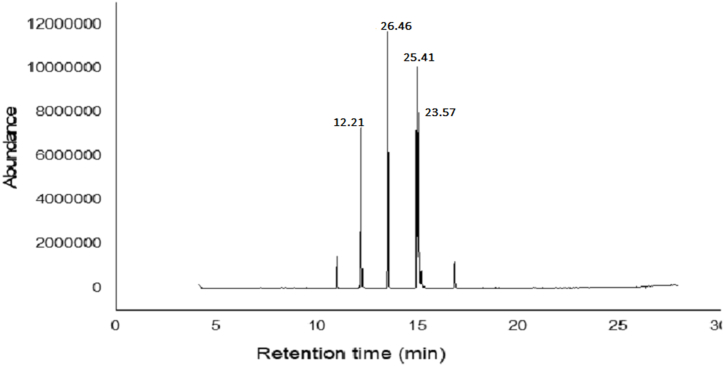
The GC-MS chromatogram of the *A. squmosa* seed.

GC-MS analysis of the hexane fractionated extract of *A. squmosa* seed revealed the presence of seven main compounds (Table 10). All compounds with qualities greater than 94 % and area percentages greater than two were identified by using the NIST 2014 mass spectral library search. The major components identified in *A. squmosa* were nonadecane (2.35 %), hexadecanoic acid meth (16.06), heneicosane (25.42 %), 9, 12-octadecadienoic acid (23.35 %), and 9-octadecenoic acid (26.466 %), and methyl stearate (2.55 %) (Fig. 5).

**Table 10:** GC-MS analysis of *Annona squmosa* seed extract.

PK	Rt	Area Pct	Compound name	Formula	Q
1	10.99	2.35	Nonadecane	C19H40	94
2	12.21	16.06	Hexadecanoic acid, methyl ester	C17H34O2	98
3	13.53	25.41	Heneicosane	C21H44	96
4	14.96	26.46	9-Octadecenoic acid (Z)-, methyl ester	C19H36O2	99
5	15.05	23.57	9,12-Octadecadienoic acid, methyl ester	C19H34O2	99
6	15.2	2.55	Methyl stearate	C19H38O2	98
7	16.84	3.56	Hexadecane	C16H34	94

Pk = peak number, Rt = retention time, area Pct. = area percentage, Q = quality.

### 3.6 GC-MS analysis of *Eucalyptus globulus* leaves

**Fig. 6:**
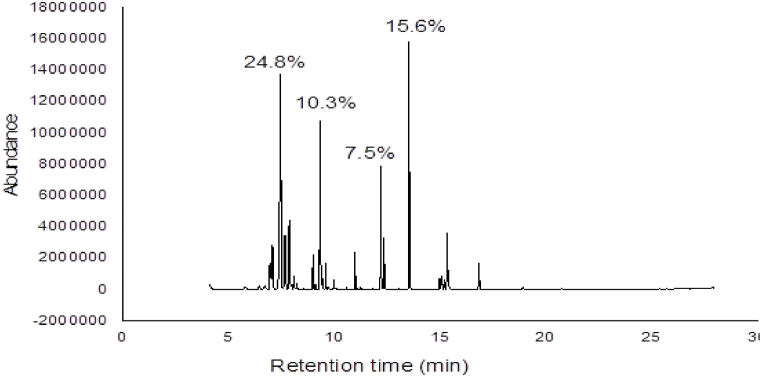
The GC-MS chromatogram of the *Eucalyptus globulus* extracts.

GC-MS analysis of hexane extract E. globulus leaves revealed the presence of 20 compounds (Table 11). Compounds with qualities greater than 80.00 % and area percentages greater than one were identified by using the NIST 2014 mass spectral library search. The names of identified compounds with their respective area percentages, retention time, compound name, and qualities are given in Table 12. The main components identified in the extracted extracts of E. globulus were aromandendrene (24.83 %), globulol (10.33 %), heneicosane (15.56 %) and hexadecanoic acid, methyl ester (7.50 %) (Fig. 6).

**Table 11:** GC -MS analysis of *Eucalyptus globulus* leaves.

PK	Rt	pc	Compound name	Formula	Quality
1	6.97	3.07	Alpha-Terpinyl acetate	C12H20O2	91
2	7.08	5.82	Gurjunene	C15H24	99
3	7.45	24.83	Aromandendrene	C15H24	99
4	7.68	5.84	Alloaromadendrene	C15H24	99
5	7.88	6.12	Viridiflorine	C15H26	99
6	8.10	1.04	Gamma-Muurolene	C15H24	87
7	8.99	1.75	Epiglobulo	C15H24	93
8	9.33	10.33	Globulol	C15H26O	99
9	9.43	0.9	Epi-γ-Eudesmol	C15H26O	85
10	9.60	1.18	Rosifoliol	C15H26O	82
11	9.99	0.66	Methyl tetradecanoate	C15H30O2	97
12	10.98	1.75	Hexadecane	C16H34	90
13	12.20	7.5	Hexadecanoicacid, methyl ester	C17H34O2	98
14	12.35	3.25	2-Heptadecanone	C17H34O	92
15	13.53	15.56	Heneicosane	C21H44	95
16	14.96	0.76	Elaidic acid	C19H36O2	87
17	15.06	1.51	Cis-9,12-Octadecadienoate	C19H34O2	95
18	15.20	0.81	Methyl stearate	C19H38O2	97
19	15.34	5.13	Linolenic acid	C19H32O2	96
20	16.84	2.21	Tricosane	C23H48	95

Pk = peak number, Rt=Retention time, Pct. = area percentage, Q = quality.

**Table 12:** GC-MS analysis of *Tagetes minuta* whole plant.

PK	Rt	Pct	Compound name	Formula	Q
1	10.99	0.62	Nonadecane	C19H40	95
2	12.1	0.6	9-Hexadecenoic acid, methyl ester, (Z)-	C17H32O2	99
3	12.21	19.1	Hexadecanoic acid, methyl ester	C17H34O2	98
4	13.53	5.78	Heneicosane	C21H44	99
5	14.97	4.38	9-Octadecenoic acid (Z)-, methyl ester	C19H36O2	99
6	15.06	62.79	Linoleic acid	C19H34O2	99
7	15.2	5.46	Methyl stearate	C19H38O2	99
8	16.84	1.28	Pentadecane	C15H32	89

Pk = peak number, RT = Retention time, Pct. = area percentage, Q = quality.

### 3.7 GC-MS analysis of *Tagetes minuta* whole plant

**Fig. 7:**
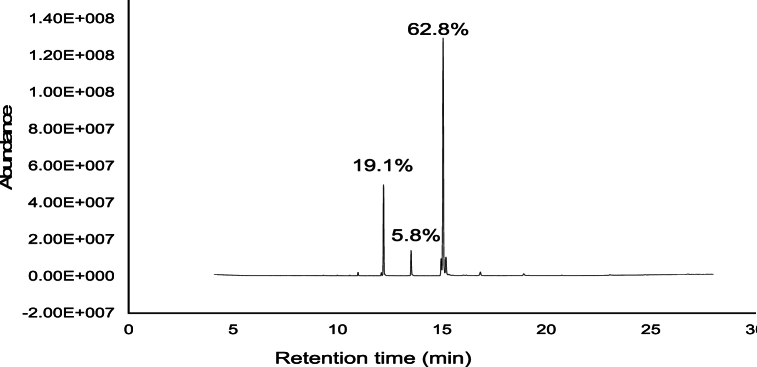
The GC-MS chromatogram of *Tagetes minuta* extracts.

GC-MS analysis of *T. minuta* whole revealed the presence of eight compounds (Table 12). All compounds with qualities greater than 89 % and an area percentage greater than one were identified by using the NIST 2014 mass spectral library search. The names of identified compounds with their respective retention times, area percentages, compound name, and qualities are given in Table 12. As shown in the table, the main components identified in the extracts were methyl 10-trans, linoleic acid (62.79 %), hexadecanoic acid, methyl ester (19.10 %), heneicosane (5.78 %), and methyl stearate (5.46 %) (Fig. 7).

## 4 Discussion

The results of the current laboratory study indicated that locally available plant materials possess insecticidal potential that can be used in the controlling of *S. zeamais* comparable to that of the standard synesthetic insecticide. All fractioned extracts from plant species admixed with maize seeds caused significant mortality in adult *S. zeamais* due to the presence of bioactive compound in the extracts. In this study, the highest mortality of 100 %, 100 % and 100 % of adult *S. zeamais* was recorded in seeds treated with all fractions of *E. globulus, T. minuta*, and *A. squamosa* at concentrations of 15 g/kg at 96 h after application of treatment, respectively. The highest concentration applied at a rate of 15 g/kg gave 100 % mortality of *S. zeamais* treated with all solvent fractions of *E. globulus* extract within 48 h after application of the treatment. The methanol fraction extracts of the *E. globulus* and *T. minuta* plant have resulted in a significant mortality rate of *S. zeamais* at a dose of 5 g/kg [27]. However, Fraction extracts of *A. squamosa* with chloroform relatively resulted in a lower mortality rate of *S. zeamais* at a dose of 5 g/kg within 48 h after treatment application compared to a fraction of *E. globulus*. This clearly indicated that insecticidal potent of plant extract directly increase when the concentration and exposure time increase [19]. Ribeiro [28] stated that Aromadendrene is a sesquiterpene hydrocarbon found from Eucalyptus species and other aromatic plants. Similarly, Sadiq [29] reported that it’s may disrupt the nervous system of insects by interfering with neurotransmitter pathways, leading to paralysis or death. It can also act as a respiratory toxin by inhibiting the insect's spiracle function, suffocating the pest.

The number of F1 progeny produced in each fraction treatment was significantly low due to the insecticidal potential of plant extracts compared to the number of progenies produced in the control. When the grains are coated with the extracts, their surfaces may deter egg-laying. The extract itself may also coat eggs and kill them by bioactive [25]. Maize seed treated with fraction hexane and fraction methanol extracts at higher extraction levels applied at 10 g/kg and 15 g/kg of grain reduced the emergence of *S. zeamais* by 100 %. Therefore, the extracts showed the presence of bioactive compounds that inhibit the life of insects [25].

The repellent action of three fractionated extracts was studied and showed that, at the highest concentration (5 and 10 g/kg), the fractionated extracts of *T. minuta* and *E. globulus* had a strong repellent action (96 ± 0.30) and (96.6 ± 0.33) against *S. zeamais*. The statistically significant differences in the interaction between the treatment of plant species, solvent, concentration, plant species and concentration and solvent and concentration (p < 0.05) (Table 7). The repellent activity of *E. globulus* and *T. minuta* progressively increases with increasing concentration against stored grain insect pests in agreement with a study by Ref. [30]. The repellent activity observed in the plant extracts tested in this study may be attributed to the presence of bioactive compounds [28].

The fumigant test of fractionated extracts derived from *E. globulus* and *T. minuta* exhibited significant toxicity against *S. zeamais*. Hiruy and Getu [31] reported that one of the most valuable characteristics of the fractionated extract is its ability to act as fumigants against insects, making them a promising option for pest control in storage facilities without the need for direct application onto the pests, this in agreement with Ribeiro et al. [32].

Several studies have investigated the efficacy of fractionated extracts against *S. zeamais*. According to Kapenga [19], the essential oil of *E. globulus* leaves against *S. zeamais* and fractionated oils was found to be effective in reducing *S. zeamais* mortality by up to 100 % at a concentration of 30 μl/L. Mossi et al. [33] reported that the essential oil of Eucalyptus species presented insecticidal and repellent properties against *S. zeamais* in line with the present study. Santos et al. [34] reported on the insecticidal activity of Tagetes species on *S. zeamais* and stated the possibility of Tagetes species extracts being a suitable alternative to the use of synthetic insecticides. A study by Ribeiro et al. [35] showed that extracts prepared from *A. mucosa* seeds in hexane and dichloromethane gave LC90 values of 259.31 and 425.15 mg/kg, respectively.

Furthermore, our results consistently indicated that the fractionated extract derived from *T. minuta* and *E. globulus* showed the highest level of repellent toxicity in all treatments. Similarly, observations have been reported that the essential oils of *E. globulus* and *T. minuta* were the most promising plants for controlling pests [16,34]. In all evaluations, the effectiveness of the treatments and the mortality rate of *S. zeamais* depend greatly on the level of application, solvent, and exposure period. Thus, researchers need to consider the above criteria in selecting effective botanicals to formulate bio-insecticides against stored grain pests [35]. The development of weevils in untreated grain was not affected and they had a high rate of feeding [10]. The results of this study could provide valuable insights into the potential of these plant extracts as natural insecticides for the protection of stored maize. The extracts of *T. minuta* generally proved to be good inhibitors of the emergence of F1 progeny and repellency; however, the hexane extract of the *E. globulus* fraction showed the maximum inhibitory properties. Finally, the *E. globulus* hexane fractions were comparatively the most effective.

The discovery of numerous bioactive compounds from different *E. globulus, A. squmosa*, and *T. minuta* hexane extracts through GC-MS analysis could be of pest control importance. The majority of active molecules in various extracts are significant compounds that are biologically active [14,36]. Among this compound, the major components identified in the extracted *E. globulus* were aromandendrene (24.83 %), globulol (10.33 %), heneicosane (15.56 %) and hexadecanoic acid, methyl ester (7.50 %). In this study, the GC–MS study revealed aromadendrene to be the most abundant compound of *E. globulus* leaves. A similar composition has also been reported by Mulyaningsih et al. [37] and Ribeiro et al. [38] Aromadendrene and globulol are key contributors to the insecticidal and repellent properties of certain essential oils. Their complementary mechanisms of action make them effective in managing a wide range of insect pests. While challenges remain, these compounds hold great potential as eco-friendly alternatives to synthetic pesticides, particularly in integrated pest management strategies.

*A. squmosa* plant extract contains active chemical constituents such as nonadecane, hexadecanoic acid, methyl ester, heneicosane, 9-octadecenoic acid (Z)-, methyl ester, 9, 12-octadecadienoic acid, methyl ester, methyl stearate and hexadecane. Among the identified compounds, the major components identified in the extract of *A. squmosa* were nonadecane (2.35 %), hexadecanoic acid meth (16.06 %), heneicosane (25.42 %), 9, 12-octadecadienoic acid (23.35 %), 9-octadecenoic acid (26.466 %), and methyl stearate (2.55 %). Zahid et al. [39] reported the presence of saturated and unsaturated fatty acids in ethanolic and n-hexane fractions of *A. squamosa* seed extract.

The extract of the *T. minuta* hexane fraction contains a few chemical compounds such as nonadecanen 9-hexadecenoic acid, methyl ester, hexadecanoic acid, methyl ester, heneicosane, 9-octadecenoic acid, methyl ester, methyl 10-trans, 12-cis-octadecadienoate, methyl stearate and pentadecane. All the major compounds from different extracts are biologically active molecules [39]. The major components identified in the extract were methyl 10-trans, methyl 10-trans, linoleic acid (62.79 %), hexadecanoic acid, methyl ester (19.10 %), and heneicosane (5.78 %), and methyl stearate (5.46 %). Linoleic acid is an abundant compound in *T. minuta* in agreement with the study by Al-Robai et al. [40]. It is worth noting that these compounds have been previously identified in several other species of plants in line with [25,41,42]. However, based on the GC-MS analysis result, the three plant species extraction has a common bioactive compound in the extract that can be used for insect pest control.

## 5 Conclusion

Generally, the results of this experiment showed that fractionated extracts of plant species were effective in controlling *S. zeamais*, comparable to Actellin 2 % dust. In this study, all fractions of *E. globulus* extracts offer a promising alternative to synthetic pesticides for pest control. The utilization of plant extracts to manage insect pests represents a sustainable, cost-effective, and environmentally friendly solution to a significant agricultural challenge. For smallholder farmers in developing regions, these findings could enhance food security, reduce economic losses, and promote healthy farming practices. Many smallholder farmers already possess traditional knowledge about using plants for pest control. Scientific validation of these botanical extracts could bridge the gap between traditional practices and modern agricultural science and encourage the adoption of scientifically informed, sustainable pest control practices. However, successful adoption will depend on addressing challenges related to production, distribution, and farmer education, more research and development is needed to optimize its efficacy and ensure its safe and sustainable use in agricultural practices.

## CRediT authorship contribution statement

**Fikadu Kifle:** Writing – review & editing, Writing – original draft, Software, Resources, Project administration, Methodology, Investigation, Formal analysis, Data curation, Conceptualization. **Melaku Girma:** Writing – review & editing, Validation, Supervision, Resources, Funding acquisition, Conceptualization. **Araya Gebresilassie:** Writing – review & editing, Validation, Supervision, Resources, Conceptualization. **Yitbarek Woldehawariat:** Writing – review & editing, Validation, Supervision, Resources, Conceptualization. **Estifanos Ele:** Writing – review & editing, Supervision, Resources, Formal analysis, Data curation, Conceptualization.

## Data availability statement

The datasets used and/or analyzed during this study are available from the corresponding author upon reasonable request.

## Declaration of competing interest

The authors declare that they have no known competing financial interests or personal relationships that could have appeared to influence the work reported in this paper.
